# Optimizing in vitro slow-growth conservation media for garlic under ambient conditions: further implications for core set accessions

**DOI:** 10.1186/s12870-025-06892-1

**Published:** 2025-08-04

**Authors:** Ashwini Prashant Benke, DalasanuruChandregowda ManjunathaGowda, Vijay Mahajan, Digambar N. Mokat

**Affiliations:** 1 ICAR-Directorate of Onion and Garlic Research, Rajgurunagar, Pune, 410505 Maharashtra India; 2 Department of Botany, Savitribai Phule Pune University, Pune, India

**Keywords:** Genetic fidelity, Molecular marker, Osmotic agents, Osmotic stress

## Abstract

**Background:**

Garlic is a bulbous crop exhibiting sufficient variations in morpho-biochemical characteristics. The entire garlic germplasm is conserved through clonal propagation at a field gene bank and thus prone to climatic changes’ effects. Therefore, maintaining the crop’s genetic diversity in a suitable alternative form is crucial. The current study standardized the protocol for slow-growth conservation of garlic (*in vitro)* at an ambient temperature. Plantlet regeneration through the shoot meristem was subjected to 21 treatments with three osmoticum, namely sucrose (1, 2, 3, and 4%), sorbitol (2 and 4%), and mannitol (2 and 4%), alone or in combination, including the control, at an ambient temperature in 2 × 21 factorial design. Furthermore, the identified treatment was validated using a core set of garlic accessions (46) to study the genetic response to in vitro slow-growth conservation.

**Result:**

A pooled analysis revealed significant differences in the response of two garlic varieties, Bhima Omkar and Bhima Purple, to varying concentrations and combinations of three osmotic agents (*p* < 0.05). Over time, substantial shooting, rooting, and plant status changes were observed. However, treatment interaction effects showed no significant variation (*P* ≥ 0.05). In the first month of conservation, treatments with sucrose alone (1% sucrose, 2% sucrose, 3% sucrose, and 4% sucrose) recorded healthier and quicker growth, whereas those with sorbitol and mannitol alone resulted in slower growth. The same growth pattern was recorded in the third month of conservation, except that the survival rate of the plantlets decreased. Media in the sucrose-only treatments dried up sooner than those in the other media combinations. At 6 months, among the 21 medium combinations tested, Murashige and Skoog’s medium supplemented with 4% sucrose and 2% sorbitol demonstrated significantly favorable delayed growth under ambient temperature conditions (25 °C ± 2), achieving a 90–92% survival rate of plantlets, outperforming other treatments. A total of 46 garlic core set accessions were cultured in the specified medium. After 1 year of conservation with two subcultures at 6-monthintervals, growth parameters, including mortality (%), plant status, and shoot and root growth, were assessed. The plant status data revealed genotypic variability, grouping the accessions into three categories containing 19, 15, and 12 genotypes, respectively. These groups were ranked in descending order based on plant status, shoot and root growth, and mortality. Genetic purity analysis using three ILP primers indicated no significant molecular-level changes.

**Conclusion:**

This protocol presents an efficient, cost-effective, and scalable approach for the slow-growth conservation of diverse garlic genotypes using osmotic agents. Beyond minimizing natural deterioration during in vivo field gene bank, it also enables the identification of osmotic stress-tolerant genotypes, which hold promise as potential parental lines in breeding programs targeting drought resilience.

**Supplementary Information:**

The online version contains supplementary material available at 10.1186/s12870-025-06892-1.

## Introduction

Garlic (*Allium sativum* L.) is the second most significant species in the *Alliaceae* family, with onion being the first one. Garlic is an annual crop predominantly grown worldwide by cloves or bulbils. It is used as a vegetable, spice, and condiment [1, 2]. China accounts for 80% of the global garlic production, followed by India. Over the past two decades, garlic production in India has increased from 497 to 2910 metric tons, whereas productivity has merely increased from 4.16 to 5.27 t/ha [3]. The low productivity is attributed to the susceptibility of garlic varieties to biotic and abiotic stresses. Viral infections are major factors causing a decline in garlic bulb quality and productivity in the subtropical zone [2, 4]. Garlic has been highly prized for medicinal uses from antiquity, and even now, the demand for garlic is substantially high in pharmaceutical industries [5]. Garlic is a rich source of a diverse array of bioactive compounds, including allicin, S-allyl-cysteine, diallyl disulfide, diallyl trisulfide, ajoene, flavonoids, phenolic compounds, and gamma-glutamyl cysteines. These compounds are directly associated with a range of health benefits, such as cardio-protective effects, immune support, anticancer properties, and antidiabetic activities [4, 5]. Garlic exhibits an immense range of variability in bulbs, leaf-related features, and yield potential, although it is sexually sterile and commonly propagated through cloves [2]. The Indian Council of Agricultural Research–Directorate of Onion and Garlic Research (ICAR-DOGR), Rajgurunagar, Pune, India, serves as a National Active Germplasm Site (NAGS). It is primarily responsible for preserving garlic germplasm diversity and conserving accessions against natural disasters [1]. ICAR-DOGR currently maintains nearly 700 garlic germplasm in the field gene bank [4], which is vulnerable to natural calamities and other challenges such as high input costs, labor, and climate change. To address these issues, in vitro slow-growth conservation methods offer a viable alternative. These methods help mitigate limiting factors, ensure steady genetic stock maintenance, and are more cost-effective than field maintenance or cryopreservation. In vitro techniques improve germplasm distribution, allow year-round access, reduce disease transmission, and facilitate virus removal *via* shoot meristem culture [6]. Methods include in vitro culture conservation, cryopreservation, and slow-growth conservation [7].

Cryopreservation and traditional conservation methods for garlic germplasm involve high costs, expert labor, and time-intensive procedures [8], making them less practical [9, 10]. Cryopreservation, while common, requires significant investment in high-end facilities [11, 12]. In contrast, in vitro slow-growth conservation offers a cost-effective and efficient alternative. By adjusting culture conditions—such as temperature, osmotic agents (e.g., sucrose, mannitol, sorbitol), growth inhibitors [13], and light intensity—this method slows multiplication rates while maintaining germplasm vigor, genetic fidelity, and low disease load. It facilitates medium-term conservation, global distribution, and rapid multiplication, making it increasingly viable for garlic and other crops like potatoes [13], grapes [14], and strawberries [15]. These osmotic agents induce an imbalance in water potential between the cell’s interior and its external environment, often triggered by changes in external conditions. This disruption in water potential results in the gain or loss of water through osmosis, leading to a breakdown in cellular homeostasis. Consequently, cells experience significant physiological and biochemical stress, impairing their normal functions [13, 15]. To date, numerous studies have focused on garlic cryopreservation [11, 12]. However, this article demonstrates a cost-effective approach for low-budget laboratories to preserve their valuable clonally propagated garlic germplasm, ensuring its long-term maintenance and accessibility.

The likelihood of somaclonal variation increases with repeated subcultures [1], making genetic fidelity testing essential for in vitro-conserved plantlets compared to the original accession. Molecular markers such as RAPD, ISSR, SSR, and ILP have been widely used for this purpose [11, 13–15], typically revealing no significant genetic differences. However, limited research has examined the varying effects of in vitro conservation on diverse and heterozygous crop populations.

This study developed an optimized in vitro slow-growth conservation medium for garlic, maintained at an ambient temperature of 25 °C ± 2 °C, using osmotic regulators. With India’s diverse garlic collection distilled into 46 core set accessions, a subsequent experiment was conducted to evaluate their response to the standardized conservation medium and protocol. Phenotypic analyses, including assessments of genetic variability across recorded traits, were performed to validate the effectiveness of this approach.

## Materials and methods

### Plant material

In the initial experiment, we tested two garlic varieties: Bhima Purple, characterized by its purple-striped, clove-skinned appearance, and Bhima Omkar, distinguished by its white clove skins. Both varieties were developed and released by the Indian Council of Agricultural Research (ICAR) at the Directorate of Onion and Garlic Research (DOGR). Using these two varieties, we standardized in vitro slow growth conservation medium supplemented with an osmoticum (sucrose, sorbitol, and mannitol) at ambient temperature and identified the best specified media suitable for slow growth conservation at ambient temperature. In the subsequent experiment, 46 core garlic accessions (Table S4) were evaluated to assess the genotypic response to the specified media identified in the first experiment for in vitro slow growth conservation. To generate the experimental material, pure seed material of two garlic varieties and 46 core set accessions were cultivated on an 8 m² plot during the winter of 2020, with a plant spacing of 10 cm and row spacing of 15 cm. Agronomic practices recommended by ICAR-DOGR were strictly followed to ensure the production of high-quality bulbs. Harvested bulbs, averaging a clove weight of 1.5 g, were utilized for meristem isolation to regenerate plantlets.

### Surface sterilization and culture medium Preparation

During the surface sanitation process, the outer papery layer of garlic cloves was carefully removed, and the cloves were rinsed under tap water for 10 min to eliminate dirt and dust particles. They were then washed twice with distilled water (5 min each) before being disinfected using 70% ethanol and 0.2% sodium hypochlorite solutions for 15 min each. All disinfection steps were performed under aseptic conditions within a laminar airflow cabinet [4]. Using a stereomicroscope (Leica MZ6), the meristem was isolated from the sterilized cloves with autoclave-sterilized forceps and a scalpel equipped with a razor blade (HiMedia). The apical meristem, measuring 0.1–0.5 mm, was excised from the basal section of the cloves and inoculated onto Petri plates containing Murashige and Skoog (MS) medium supplemented with 0.1 mg/L Naphthyl Acetic Acid (NAA) and 1.0 mg/L kinetin [6, 16].

After three weeks of culture, shoot tips emerged from the meristem tissues. These shoot tips were transferred to Gamborg B5 medium enriched with 3% sucrose to support the development of healthy plantlets with well-formed roots [4]. After an additional three weeks, the fully developed plantlets, characterized by a height of 10–12 cm and root length of 1–2 cm, were subjected to osmotic stress treatments as detailed in Table S1.

The described protocols for surface sterilization, meristem inoculation, and plantlet regeneration were consistently applied to prepare initial plantlets for both the garlic varieties and the 46 core set accessions [4].

In total, 21 treatments were formulated using three osmotic agents, namely sucrose (1, 2, 3, and 4%), sorbitol (2 and 4%), and mannitol (2 and 4%), alone or in combination(Table 1), including the control. The experiment was conducted in a 2 × 21 factorial design. In the first experiment, the in vitro established plantlets (regenerated from the shoot meristem) of garlic varieties (Bhima Purple and Bhima Omkar) were independently cultured on MS media containing varying 21 combinations and concentrations of sucrose, sorbitol, and mannitol, including the control (media without any osmoticum). A total of seven replicates were maintained for each treatment. Here, a single plantlet was cultured in each test tube, which was then sealed with parafilm tape (HiMedia) and a polypropylene lid. Similarly, seven plantlets in each treatment were considered as seven replicates. The combination found to be most suitable for slow-growth conservation at an ambient temperature (25 °C ± 2) was used to evaluate the genotypic response of the 46 core set accessions of garlic [4]. Therefore, in the subsequent second experiment, the established in vitro plantlets of the 46 accessions were cultured using a RBD on the standardized combination (identified from the aforementioned 21 treatments), which was MS medium supplemented with sucrose 4% + sorbitol 2%,for slow-growth conservation studies. Further, we also assessed the genetic fidelity of representative core set accessions conserved in specified media along with control plantlets (in vitro plantlets grown on Gamborg B5 media) using three ILP markers [2].

**Table 1:** Analysis of variance of garlic varieties (2) over the 21 osmotic treatments for 1^st^, 3^rd^ and 6^th^ month of conservation period

Source	Degree of Freedom	Mean Square				
		Shoot length (cm)	Root Length (cm)	Number of leaves	Number of root	Survival (%)
Replication	6	0.157	0.694	0.013	0.086	0.064
Treatment	20	8.625**	4.284*	0.559**	1.787**	1.341**
Varieties	1	3.520	50.870	0.394	22.933	0.424
Period	2	10.022*	29.721*	12.630*	6.090*	30.395*
Var*Period	2	0.202**	3.267*	1.255*	4.814*	0.393*
Var*Trt	20	2.132*	1.394*	0.248*	0.588*	0.157*

The asterisks (*) next to the mean square values indicate the statistical significance of the observed differences based on ANOVA. * (Single asterisk): Significant at the 5% level (p ≤ 0.05)-;This means there is a less than 5% probability that the observed variation is due to random chance.** (Double asterisk): Significant at the 1% level (p ≤ 0.01)-There is less than 1% probability that the observed effect is due to chance. The result is highly significant

Before adding agar to media (21 treatments), the pH was adjusted, and the media were autoclaved at 121 °C for 20 min. Cultures were incubated in a growth chamber at 25 °C under a photoperiod of 16 h of light (3000 lx) and 8 h of darkness [16]. The experiment utilized MS and B5 media (Dusefa), growth hormones (Sigma Aldrich), osmoticum (HiMedia), and chemical reagents (HiMedia) [4].

### Genetic fidelity test using ILP molecular markers

At 6^th^ month after in vitro slow-growth conservation at an ambient temperature, the leaves of randomly selected plantlets of the fifteen core set accessions were collected and stored at − 80 °C. The DNeasy plant micro kit was used to extract whole genomic DNA (Thermo Scientific) [4] UV–V is spectrophotometry (Nanodrop TMND2000c) and agarose gel analysis were performed to assess DNA purity and integrity. To ensure repeatability, PCR amplification was performed with Intron Length Polymorphism (ILP) markers [17]. A single PCR mixture contained 25 ng genomic DNA (1µL), 10× PCR buffer which contains 1.5 mM MgCl_2_, 2.5 mM dNTP mix, 0.8µL each sense and antisense primers, and Taq DNA polymerase. The reaction mixture was loaded on the ProFlex PCR system (Applied Biosystems byThermo Fisher Scientific, USA) in 96-well blocks. The thermal cycler program included 1cycle of 94 °C denaturation for 3 min, 30 cycles of PCR amplification by using the step protocol (denaturation at 94 °C for 1 min, annealing at 52.3 °C for 1 min, and polymerization at 72 °C for 2 min), and a final extension at 72 °C for 10 min. The amplified product was stained with ethidium bromide (0.5 µg/ml) in 2% agarose (HiMedia) prepared in 1X TAE (Tris-Acetate-EDTA) buffer. The gels were visualized in a UV transilluminator (Genei) and photographed using a gel documentation system (Bio-rad) [4]. Three ILP primer pairs were used for the genetic fidelity test (Table 4).

### Statistical analysis

Plantlet growth and its condition during in vitro conservation are dynamic. Thus, the overall health of the plant leaves, referred to as the plant status, was assessed based on phenotypic analysis and categorized into three distinct groups after in vitro conservation: (1) yellowish, desiccated leaves with necrotic spots, assigned a value of 1; (2) whitish-yellowish leaves, assigned a value of 2; and (3) dark green leaves with a healthy appearance, assigned a value of 3. These classes were considered cardinal numbers for further statistical analysis. Other phenotypic observations recorded for each plantlet were the average shoot length (cm), average root length (cm), leaf number, root number, and survival rate (%). The Proc ANOVA algorithm of SAS 9.3 was used to perform analyses of variance for two varieties in the first experiment [1, 4]. The recorded data on the plantlets (21 treatments) were statistically examined using the ICAR-Indian Agricultural Statistics Research Institute (IASRI) website’s online statistics portal [4]. The software JMP 10.0 was used for analyzing the means and analysis of variance for the core set accessions (46) [4] and for further clustering on genetic distance means. The 6 months was considered the threshold time for evaluating all treatments under slow-growth conservation of garlic at an ambient temperature, because this period is followed by increased mortality or the profuse growth of leaves from some parts of the plants.

## Results

### Effect of sucrose, sorbitol, and mannitol on slow-growth conservation

To standardize the protocol for slow-growth conservation using media supplemented with osmotic agents at ambient temperature (25 ± 2°c), we utilized well-established in vitro plantlets derived from the shoot meristem of garlic varieties Bhima Omkar and Bhima Purple as the initial material. The analysis of variance for varieties, treatments revealed a significant impact on growth parameters, but interaction showed (variety x treatment) for dependent traits *viz*. shoot length (cm), root length (cm), number of shoots, number of roots and survival percentage (Table 1,Table S2(a, b), Table S3(a, b)) showed non -significant difference among various growth parameters.—and the conservation period (Table 1; Table S2(a, b); Table S3(a, b)).

During the initial conservation period (30 days to culture [DTC] or one month), the full-strength MS medium supplemented with mannitol (2% and 4%) alone significantly reduced shoot and root growth parameters (Table S3a) compared to the medium supplemented with sorbitol (2% and 4%) alone or sucrose alone (Table S3a). Additionally, in the control treatment, where plantlets were grown in medium without osmotic agents, necrotic symptoms appeared after two weeks of culturing. At 30 days after transfer to culture (30 DTC), plantlets exhibited rapid growth across all sucrose concentrations (1%, 2%, 3%, and 4%), with significantly higher growth observed at the higher concentrations (3% and 4%) compared to other treatments. During this initial phase of rapid growth, media in the sucrose-only treatments dried out faster than those in other treatment combinations, impacting plantlet survival in the subsequent 90 DTC (third month of conservation). As a result, treatments containing sucrose (1%, 2%, 3%, and 4%) were effective in maintaining plantlets in a healthy state for up to 30 days of culturing (one month), with 100% survival. (Table S3a; Fig S1). However, due to the rapid and vigorous growth induced by sucrose supplementation, sucrose alone was unsuitable for prolonged slow growth conservation. During the first month of conservation (30 DTC), plantlets cultured with sorbitol or mannitol alone (2% and 4%) exhibited significantly reduced growth parameters (*p* < 0.05) compared to those cultured with sucrose alone, which maintained survival rates of 66–80%. Growth rates were significantly lower in the mannitol-alone treatments than in other combinations, indicating a pronounced inhibitory effect on plant development.

At 90 DTC, mannitol-alone treatments (2% and 4%) exhibited the smallest increases i.e. non-significant in measured growth parameters (*p* < 0.01), with survival rates reduced to 65%. Sorbitol-alone treatments at 2% and 4% concentrations showed statistically significant reductions in survival rates to 50% and 80%, respectively. In contrast, treatments with sucrose alone (1%, 2%, 3%, and 4%) led to a highly significant reduction in survival rates, ranging from 28 to 34% (*p* < 0.001), along with a marked decline in plant health compared to their condition at 30 DTC (Table S3b). However, combination treatments of sucrose and sorbitol resulted in the highest survival rates (85–100%, *p* < 0.05) and significantly improved plant health scores (*p* < 0.01) compared to other treatments. (Table S3b; Fig. S1). Beyond the third month (90 days) of culture, plantlet viability declined across treatments, with some plantlets exhibiting complete drying of leaf tips, while others formed mini bulbs at the base.

**Fig. 1:**
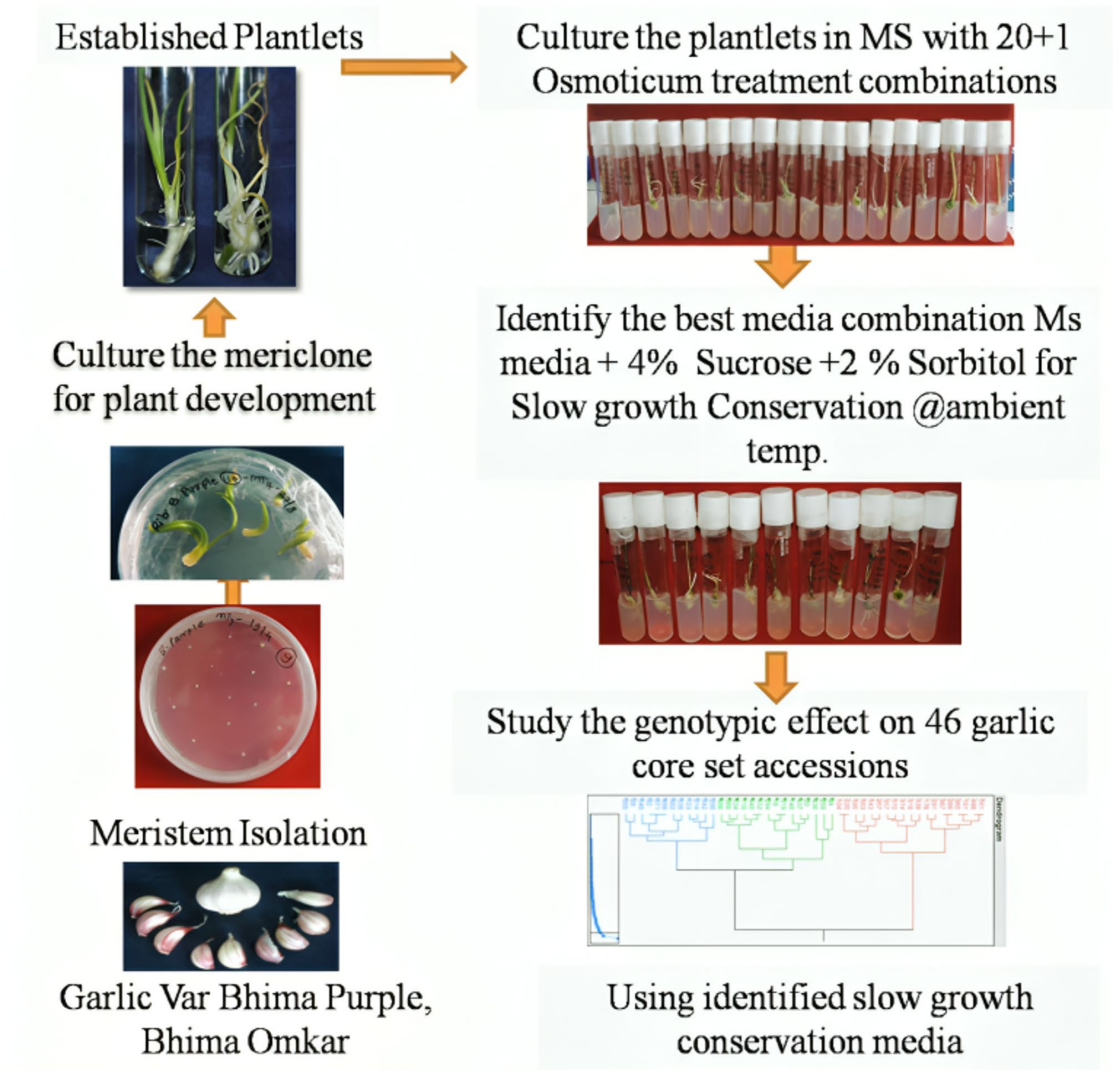
In vitro slow growth conservation strategy for garlic using meristem culture. The process begins with the selection of garlic varieties (Bhima Purple and Bhima Omkar), followed by isolation of meristems and development of elongated mericlones. These are further established into plantlets. The established plantlets are cultured in 20+1 osmoticum combinations, identifying MS + 4% sucrose + 2% sorbitol as the best medium for slow-growth conservation at ambient temperature. The optimized medium was used to screen 46 garlic core set accessions for genotypic responses

By the end of the sixth month of the conservation period, a survival rate of 0% (*p* < 0.001) was recorded for all sucrose-alone and sorbitol-alone treatments. In contrast, the mannitol-alone treatments at 2% and 4% concentrations achieved survival rates of 50% and 80% (*p* < 0.05), respectively. Notably, mini bulblets were observed in the 4% mannitol (Table 2). Additionally, plantlets exhibited significant drying and yellowing of leaves, indicating stress under prolonged conservation conditions (Fig 1).

**Table 2:** Effect of different osmotic concentrations on morphological traits of garlic at sixth month (180 Days) of in-vitro slow growth conservation of garlic plantlets

Sr no	Treatment combinations	Parameters recorded at 6th month of slow growth conservation of garlic plantlets						
		Shoot length (cm)	Root length (cm)	Number of leaves	Number of roots	Plant status	Description	Survival rate (%)
1.	Control*	-	-	-	-	-	both root and shoot dried	0e
2.	1% Sucrose*	-	-	-	-	-	bulb with dried leaves	0e
3.	2% Sucrose*	-	-	-	-	-	buil with dried leaves	0 e
4.	3% Sucrose*	-	-	-	-	-	both root and shoot dried	0 e
5.	4% Sucrose*	-	-	-	-	-	both root and shoot dried	0 e
6.	2% Sorbitol	13.25 f	0.00 e	0.00 fg	0.00 f	1.38gf	bulb formed but leaf tips are dried and the root become brown	0e
7.	4%Sorbitol	12.33 d	6.43 bc	2.00 bdc	3.00 bdc	1.80bac	bulb formed but leaf tips are dried and the root become brown	0 e
8.	2% Mannitol	10.14 bdc	5.86 bac	1.57 dc	2.57 d	1.49ef	very slow growth, plants dried after 3rd month	51 e
9.	4% Mannitol	10.57 bdac	4.71 bc	1.00 de	3.00 dc	1.54edf	very slow growth, plants dried after 3rd month	80 e
10.	1% Sucrose + 2% Mannitol	14.57 bac	7.57 bac	1.29 dc	3.57 bac	1.45f	elongated leaves and root, bulb formation	71 e
11.	1% Sucrose + 4% Mannitol	11.57 dc	5.57 ba	1.29 bdc	2.57 bdc	1.69edc	medium height green leaves, bulb formation	71 abc
12.	2% Sucrose + 2% Sorbitol	15.57 ba	6.29 bac	1.00 bdac	4.00 bdc	1.72bdc	elongated leaves and root, bulb formation	71 ab
13.	2% Sucrose + 4%Sorbitol	14.57 bdac	7.57 bc	1.29 bac	3.71 bac	1.91ba	elongated leaves and root, bulb formation	85 a
14.	3% Sucrose + 2% Sorbitol	15.14 bac	5.43 c	1.14 bac	4.43 a	1.97a	elongated leaves and root, bulb formation	90 a
15.	3% Sucrose + 4% Sorbitol	12.43 bdac	8.29 a	1.43 ba	4.29 bac	1.91ba	dark green leaves, healthy plant	85 ab
16.	4% Sucrose + 2% Sorbitol	13.47 bac	10.43 ba	1.71 a	3.29 bdc	1.83bac	elongatedgreen leaves, healthy plant	94 a
17.	4% Sucrose + 4% Sorbitol	12.00 dc	6.14 bc	1.00 bdac	4.00 bdac	1.98a	green leaves and bulb formation	98 ab
18.	2% Sucrose + 2% Mannitol	11.86 bac	7.29 bac	1.29 bdc	4.00 bdc	1.55edf	medium height green leaves, root tips brown	71 abc
19.	2% Sucrose + 4% Mannitol	13.00 bac	6.71 bac	1.14 bac	3.43 ba	1.47f	healthy green plant with bulb formation	57 abc
20.	4% Sucrose + 2% Mannitol	13.00 a	5.57 bac	1.14 bac	3.43 bdc	1.94a	dried tip of leaf, bulb formation	14 ab
21.	4% Sucrose + 4% Mannitol	12.86 bdac	5.71 bac	1.43 bac	4.14 d	1.78bac	dried tip of leaves abd bulb formation	14 ab

(Data was anaysed by squreroot transformation using SAS 9.3 Proc GLM, Duncan’s Multiple Range Test, and letters used to differentiate among treatments; Means with the same letter (s) are not significantly different by Duncuan’s Multiple Range grouping @0.05Probability) (* these treatments showed dried or dead plants leaf and roots); Root length was measured by taking representative plant out of test tube

**Fig. 2:**
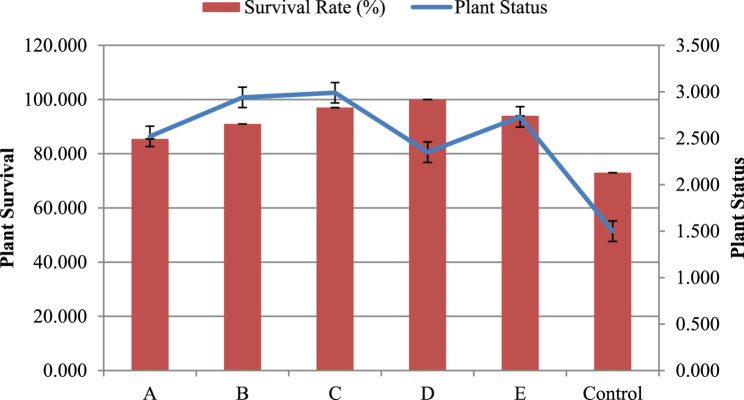
Effect of promising osmoticum treatments on plantlet survival rate (%) and plant status (1,2,and 3 categories) at 6th month of slow growth conservation at ambient temperature [A-2%Sucrose + 4%Sorbitol, B- 3%Sucrose + 2%Sorbitol, C-4%Sucrose + 2%Sorbitol, D- 4%Sucrose + 4%Sorbitol, E- 3%Sucrose + 4%Sorbitol; Plant Status: 1: yellowish desiccated leaves with necrotic spots, 2: whitish-yellowish leaves and 3: dark green leaves with a healthy appearance]

Among the combination treatments, the shortest shoot length (11–12 cm) was observed in the 1% sucrose + 4% mannitol treatment, representing a statistically significant reduction compared to other combinations (*p* < 0.05). In contrast, the 2% sucrose + 2% mannitol and 4% sucrose + 4% sorbitol treatments produced average root lengths of 5–7 cm and an average plant status rating of 1.65, indicative of moderate plant health (Table 2; Fig. 2). Notably, the 3% sucrose + 4% sorbitol treatment resulted in the longest roots (10 cm, *p* < 0.01), followed by 4% sucrose + 2% sorbitol (8 cm), 2% sucrose + 4% sorbitol (7.5 cm), and 2% sucrose + 2% mannitol (7 cm).The highest plant status scores were observed in the 3% sucrose + 2% sorbitol (1.97) and 4% sucrose + 4% sorbitol (1.98) treatments, reflecting statistically significant improvements in plant health (*p* < 0.01). Plants in these treatments exhibited dark green foliage, glossy leaves, and well-established roots. Survival rates for these combinations were also remarkable, with 85% survival for 2% sucrose + 4% sorbitol, 90% for 3% sucrose + 2% sorbitol, 94% for 4% sucrose + 2% sorbitol, and 98% for 4% sucrose + 4% sorbitol (Fig. 2). When comparing survival rates with plant health parameters, the 4% sucrose + 2% sorbitol treatment emerged as the most effective for the slow-growth conservation of garlic over six months. This treatment demonstrated superior plant health, characterized by optimal shoot length, root number, root length, and vibrant leaf color, alongside a significantly higher survival rate (*p* < 0.01). These results underscore the efficacy of tailored sucrose and sorbitol combinations in enhancing both plant health and conservation outcomes.

### Screening of garlic core set accessions

The core set of garlic genotypes (46 accessions) was successfully conserved under slow-growth conditions using a standardized culture medium containing 4% sucrose + 2% sorbitol for one year with two sub-culturing intervals. Data on shooting status, rooting behavior, and overall plantlet status were collected after culturing the initial plantlets for a year. Statistical analysis revealed that, except for mortality percentage and plant status, most other parameters exhibited non-significant differences among the core set accessions (*P* ≥ 0.05, Table S4). Pooled mean values of all parameters were utilized for cluster analysis of the 46 garlic core set accessions, providing a comprehensive evaluation of their responses under slow-growth conditions [4]. Screening with the 4% sucrose + 2% sorbitol combination, deemed to be optimal based on prior testing of 21 combinations, highlighted variability in mortality rates across the accessions. The maximum mortality rate (50%) was observed in accession DOGR-32, while the minimum mortality rate (5%) was recorded in accession DOGR-561 (Table S4, Table S5). These results underscore the suitability of the 4% sucrose + 2% sorbitol medium for the slow-growth conservation of diverse garlic genotypes, while also identifying accessions with varying sensitivities to the standardized protocol.

**Fig. 3:**
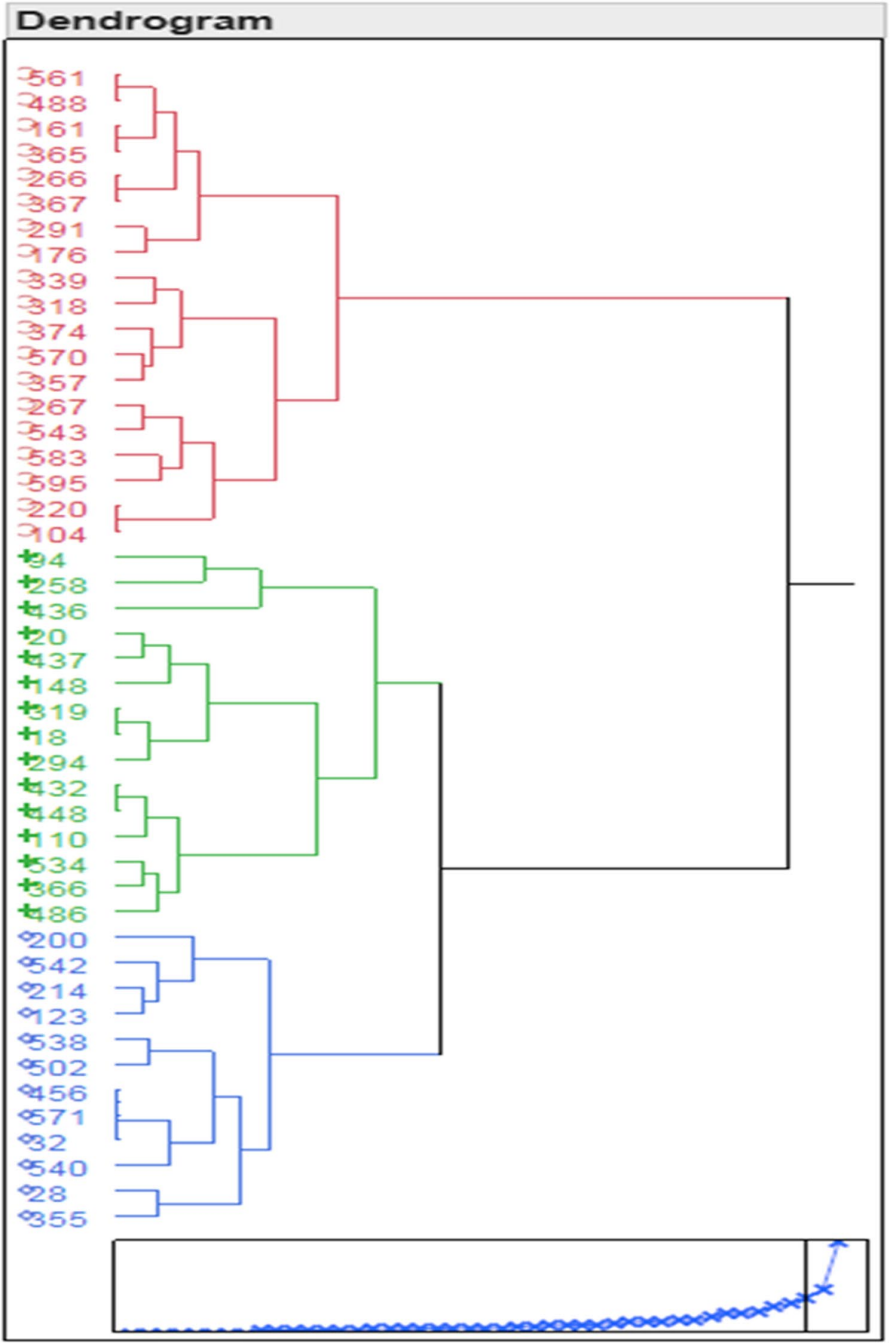
Clustering of garlic core set accessions (46) based on genetic distance revealed through characterization of slow growth conserved (in vitro) plantlets for one year with two sub culturing. The mean values of traits* viz.*, shoot length, root length, number of leaves, plant status and survival per cent were used to formulate the dendrogram through JMP 10.0 software

**Fig. 4:**
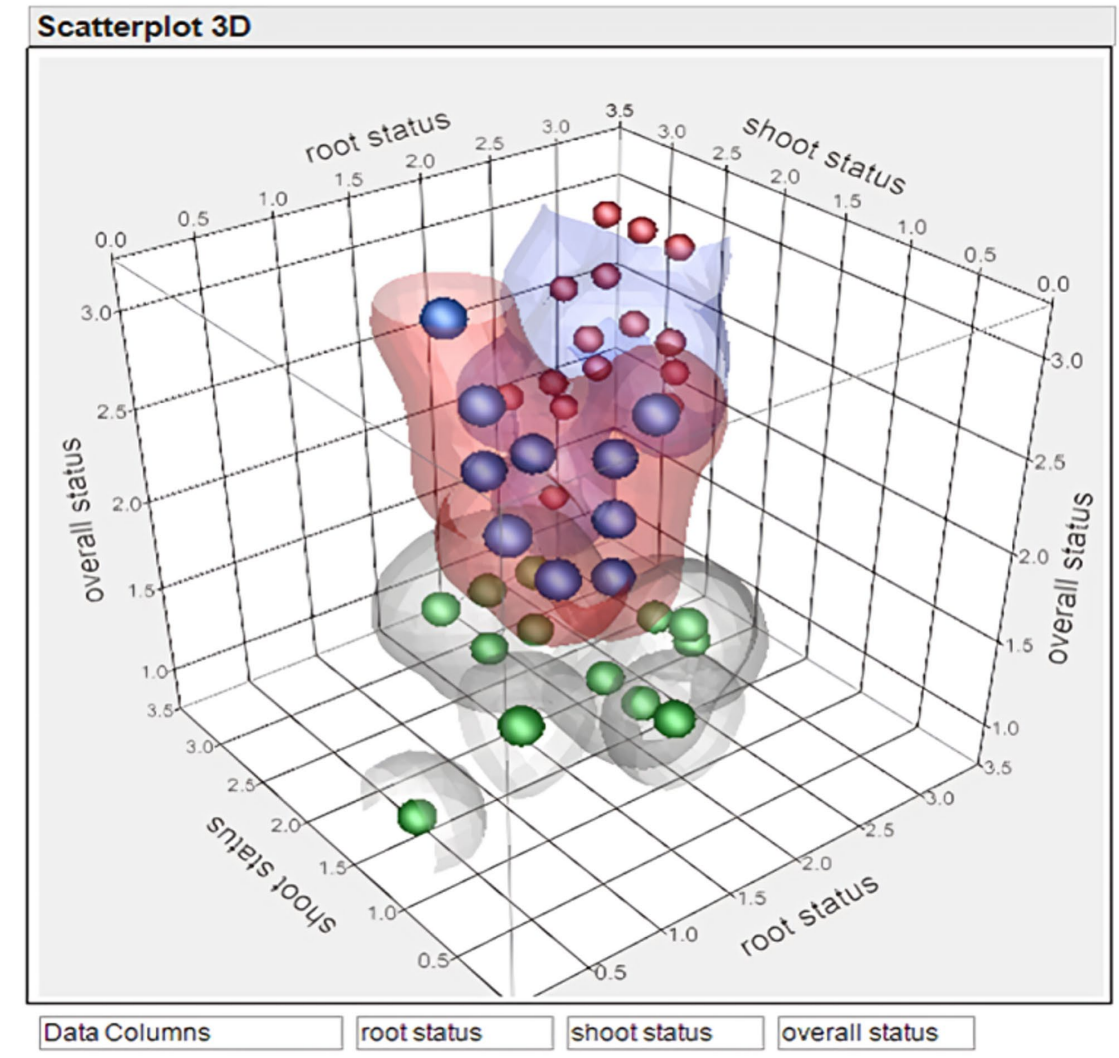
Scatter plot 3D diagram showing grouping of garlic core set (46) genotypes based on scores recorded for root, shoot and overall plant health. (red dots- first group, blue dots- second group, green dots- third group). This grouping is in coordination with formulated dendrogram

Based on the genotypic responses of the 46 accessions, the entire set was divided into three distinct groups (Figs. 3 and 4). Group 1: This group included 19 accessions (561, 488, 161, 365, 266, 367, 291, 176, 339, 318, 374, 570, 357, 267, 543, 583, 595, 220, and 104) with a ranking of 3–2.5 for shooting, rooting, and overall plantlet status, and no recorded mortality. These plantlets displayed dark green leaves, robust strength in shoots, and limited shooting and rooting. Group 2: Comprised of 15 accessions (94, 258, 436, 20, 437, 148, 319, 18, 294, 432, 448, 110, 534, 366, and 486), this group exhibited average performance across all recorded traits. Group 3: Included 12 genotypes (200, 542, 214, 123, 538, 502, 456, 571, 32, 540, 28, and 355) that showed poor performance in all traits. Plantlets in this group showed deteriorated overall growth as the conservation period progressed.

The overall mortality rate among the core set genotypes ranged from 0 to 50%. Mortality was highest in group 3 (20–50%), moderate in group 2 (5–20%), and lowest in group 1 (< 5%) (Figs. 3 and 4; Table 3; Table S4). The conservation medium supplemented with 4% sucrose and 2% sorbitol successfully preserved all garlic accessions, with those in the first group demonstrating the best genetic aptitude for long-term conservation (Table S5).

**Table 3:** Group-wise mean performance of growth parameters of garlic plantlets under in vitro slow growth conservation

Group (No. of genotypes)	Shoot length (cm)	Number of roots	Root length (cm)	Plant status	Average mortality (%)	Range of mortality (%)
I(19)	12.65 ± 0.34	3.68 ± 0.30	6.46 ± 0.74	2.91 ± 0.41	7.54 ± 1.38	5–10
II(15)	10.44 ± 0.58	2.51 ± 0.62	5.35 ± 0.84	2.49 ± 0.24	15.85 ± 2.81	10–20
III(12)	9.83 ± 0.32	2.61 ± 0.37	5.11 ± 0.54	1.43 ± 0.25	30.72 ± 8.68	20–50

(Plant status is overall plant growth referred as:1: as yellowish desiccated leaves with necrotic spots, 2: as a whitish-yellowish leaves, and 3:as a dark green leaves with a healthy appearance in a phenotypic analysis;)

This optimized medium composition is pivotal in conserving garlic genotypes under slow-growth conditions under short-term conservation, Furthermore, genotypes with a strong capacity to withstand osmotic stress—particularly those in the first cluster—represent a valuable genetic resource for future breeding initiatives focused on enhancing conservation strategies and stress tolerance in garlic.

### Genetic fidelity study using ILP markers

The primary function of any gene bank is to preserve the genetic integrity of clonally propagated crops during slow-growth conservation, which requires a standardized tissue culture protocol. In this experiment, a combination of 4% sucrose + 2% sorbitol was identified as the optimal osmotic treatment for slow-growth conservation. To assess the genetic stability of in vitro plantlets conserved using this protocol, DNA-based molecular markers were employed. Molecular analysis confirmed that all genotypes maintained in the standardized slow-growth conservation medium were genetically stable and pure. For this assessment, 15 samples were randomly selected for PCR amplification using three ILP primers [17] (Table 4), including a control sample with three experimental replicates. The ILP markers generated 2,500–2,700 amplification products, calculated based on the number of micro plants, the number of bands produced by four primers, and the three experimental repetitions.

**Table 4:** Details of ILP primers used for genetic fidelity testing of garlic clones generated through in vitro conservation

Sr. No.	Primer name	Sequence 5’ to 3’	Tm (°C)
1	AcPIP 39 F	TATGCATTTGCTCCGGCTAT	55
	AcPIP 39 R	CCAAATATATGTAGCTTCTCTCGTAAA	58
2.	AcPIP 42 F	CAGTGGTCATATTCCCAGTGAAC	60
	AcPIP 42 R	AACAGATTCCAATGTTTCTCTTAGC	58
3.	AcPIP 103 F	TCCCTAAGAAGAATCGTCAAGA	56
	AcPIP 103 R	GACATCAATCAATGGGTGCTT	55

**Fig. 5:**
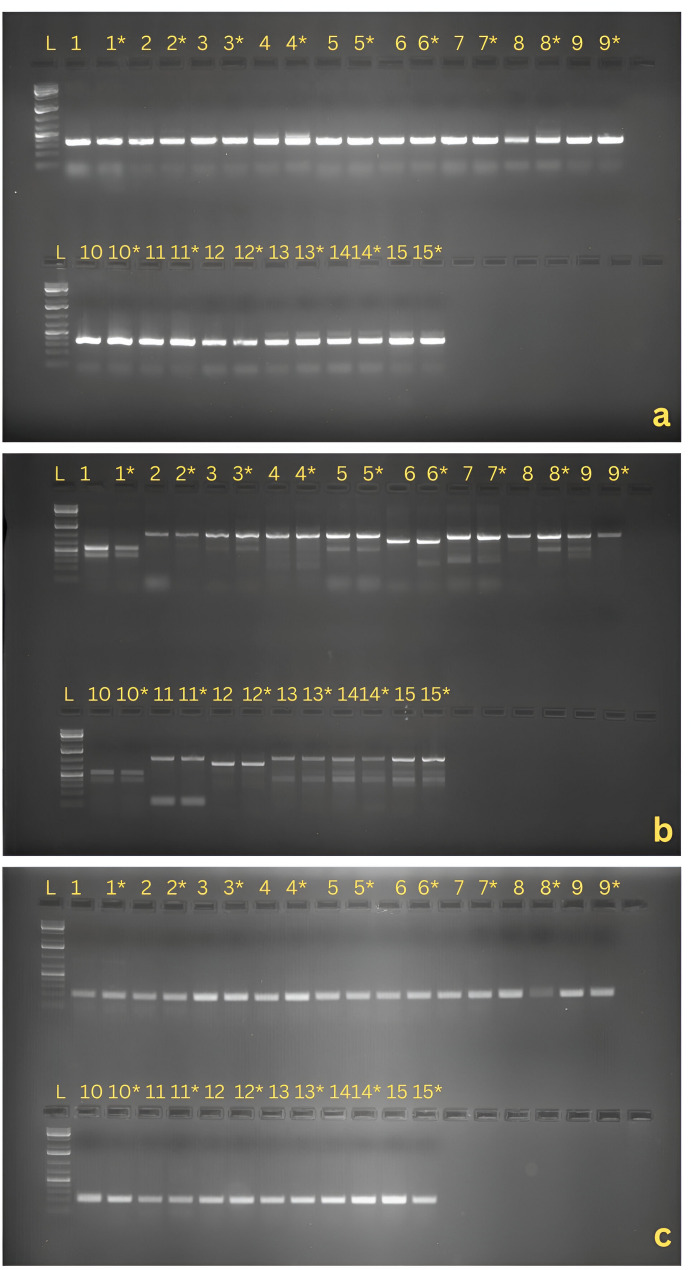
Genetic fidelity testing of randomly tested in-vitro slow growth conserved garlic samples in identified medium using Intron Length Polymorphic (ILP) markers. Amplification of ILP markers a) AcPIP 39, b) AcPIP 42, c) AcPIP 103. Legands are M- DNA ladder marker (1kb), clones are 1 & 1*-161, 2 & 2*-260, 3 & 3*-365, 4 & 4*436, 5 & 5*-571, 6 & 6*-200, 7 & 7*-123, 8 & 8*-287, 9 & 9*-339, 10 & 10*-561, 11 & 11*-401, 12 & 12*-488, 13 & 13*-148, 14 & 14*-542, 15 & 15*-273. * In-vitro clones. (Uncropped separate gel images of each primer set attached as supplementary file Fig S2, S3, S4) {1kb DNA ladder (Thermo Scientific) has been used as reference for amplicons size measurement}

The ILP fingerprints were identical across all control and conserved in vitro plantlets (Fig. 5), demonstrating that no genetic variation occurred in any of the randomly selected shoots from the 15 in vitro slow growth conserved core set accessions. These results validate the efficacy of the 4% sucrose + 2% sorbitol medium in maintaining genetic stability during long-term conservation of garlic genotypes.

## Discussion

Garlic is a non-seeded bulbous spice crop cultivated for its distinct flavor and therapeutic properties. In addition to clonal proliferation, garlic undergoes substantial genetic variation, which is reflected in its physical traits [1, 4]. As the National Active Germplasm Site (NAGS) for garlic, the ICAR-DOGR is tasked with the primary responsibility of conserving the genetic diversity of the crop while safeguarding it against the adverse effects of severe climatic conditions. Various medicinal plant species, such as *Bacopa monnieri* [18], *Pichro rhizakurroa* [12], and *Withania somnifera* [19], have been mass propagated using in vitro techniques for germplasm conservation.

Numerous researchers involved in plant conservation have reported favorable results with low-temperature conditions and cryopreservation techniques [9], particularly for long-term garlic conservation [11, 12]. However, the present study employed a combination of ambient temperature and osmotic agents to extend the subculture period, achieving a high survival rate of 85–95% after six months. This strategy proves to be particularly effective in subtropical laboratories where limited funding may constrain the use of low-temperature conservation facilities.

Performing two sub-culture cycles per year at ambient temperature is a cost-effective alternative to maintaining low-temperature storage systems. Furthermore, the addition of 3% sucrose to the basal B5 medium effectively restores plantlets to their normal condition. However, a potential limitation of this strategy is the indirect selection pressure exerted by sub-culturing, which may lead to somaclonal variation and alterations in the genetic makeup of the plantlets. Continuous culture maintenance has been shown to induce somaclonal rearrangements, polyploidy, and single-locus mutations [20], with the risk of genetic variation increasing with each subsequent subculture. For example, *Nepenthes khasiana* exhibited a genetic variation increase from 5.65% in the first generation to 7.77% and 10.87% in the second and third generations, respectively [21].

Recent studies indicate that cryopreservation or slow-growth conservation techniques reduce the frequency of mutations, with genetic variation becoming an extremely rare event under such conditions [9].Osmotic agents, by inducing osmotic stress in plantlets under conservation, function as growth retardants. When incorporated into the growth medium, these carbohydrates reduce the water potential, thereby limiting water availability to the explants [9]. Osmotic stress, induced by agents like sorbitol and mannitol, slows plant growth by altering osmotic pressure, which in turn reduces the absorption of minerals by plant cells [11, 20]. In this study, sucrose, sorbitol, and mannitol were used as osmotic agents to extend the shelf life of garlic plantlets developed in vitro. The complexity of carbohydrate structures influences the rate of energy release to the in vitro plantlets, with more complex structures resulting in slower energy release. When sucrose was used as an osmotic agent, the plantlets exhibited dark green leaves and rapid growth for a short duration before the medium dried out. Sucrose, as noted by Camillo and Scherwinski-Pereira [22], is well-suited for maintaining plant quality under in vitro conditions. In comparison to sucrose-treated plantlets, those treated with sorbitol showed reduced growth patterns. Similar findings were reported by Hassan and Bekheetal [15], who observed that, when compared with sucrose, sorbitol reduced the plant height and overall vigor of strawberry plants, while simultaneously improving survival rates. Additionally, after one year of conservation, the highest survival percentage for *Deutzia scabra* shoot tips was observed in the control group (25 g/L sucrose without mannitol at 24 °C), whereas the lowest survival was recorded in explants incubated at 4 °C [23].

Compared to sucrose treatment, both sorbitol and mannitol resulted in stunted growth, consistent with the findings of Camillo and Scherwinski-Pereira [22], who reported reduced growth in oil palm when sucrose was used. Tehrim and Sajid [14] also observed a decline in growth in grape germplasm as osmoticum concentrations increased. They noted that, unlike sorbitol, mannitol had a positive effect on the growth of the evaluated accessions. In our experiment, mannitol demonstrated a favorable conservation impact during the first three months compared to sorbitol and sucrose. However, the plantlets began to dry out by the fourth month. Previous studies have shown that all treatments involving mannitol slowed the growth of *Indigofera tinctoria*, with 10 g/L mannitol being the optimal concentration for maintaining culture survival for up to 28 weeks without the need for sub-culturing [24]. According to Lata et al. [25], concentrations of 2–4% mannitol were insufficient for the successful conservation of *Podophyllum peltatum L.* Ultimately, the optimal mannitol concentration for conservation is species-specific; however, it is important to note that higher mannitol concentrations may lead to necrosis in certain species.

Osmoticum combination treatments consistently outperformed all other treatments in terms of plant condition and survival. Throughout the study, the two varieties, *Bhima Purple* and *Bhima Omkar*, showed no significant differences in the average shoot length, root length, number of shoots, number of roots, and overall plant status. However, significant differences were observed in the average shoot length between plants treated with various osmotic concentrations and their combinations. Notably, the 4% sucrose + 2% sorbitol combination resulted in the highest survival rate (up to 90%) and the best plant status at the sixth month of conservation (Table 2).

In all treatment combinations, the morphological behavior of the plantlets remained consistent, with no abnormalities observed. In terms of conservation duration, the highest survival rates (100%) were recorded in the initial months, but survival steadily declined as the conservation period extended from 3 to 6 months. In treatments lacking carbon sources (sorbitol, mannitol, and sucrose), morphological abnormalities were observed, and *Epidendrum chlorocorymbos* plantlets exhibited poor development, accompanied by necrotic symptoms [26]. However, compared to sucrose, treatments with sorbitol or mannitol (2% and 4%, respectively) alone resulted in slower plant development at 30 days post-culturing, but with overall good plant status.

Analysis of the conservation medium’s effect on the 46 garlic core set accessions revealed differential expression in various morphological traits and survival percentages across osmotic medium combinations. All genotypes exhibited distinct genetic responses to osmotic stress, with notable variations in survival rates and other physical characteristics. The observed differences underscore the necessity of further biochemical and molecular investigations to identify garlic genotypes resistant to osmotic stress. Because identifying osmotic stress-tolerant genotypes in vitro is easier than that in the field, it is an urgent need.

Several accessions were screened for drought tolerance and their responses to water stress during callus induction and plant regeneration stages. Similar studies have been conducted in other crops, such as wheat [27], maize [13], and potato [7]. Our results related to differential expressions in the recorded traits among the genotypes are consistent with those of the aforementioned experiments. Consequently, stress-tolerant garlic accessions will be selected. Studies on osmotic stress responses have been extensively reported in rice [28], maize [13, 29], and wheat [27, 30]. These investigations encompass antioxidant systems, enzyme activities, and transcriptomics analyses, offering comprehensive insights into the molecular mechanisms underlying drought and water-logging tolerance in both tolerant and susceptible genotypes.

A key mandate of any gene bank is to maintain the genetic purity of clonally propagated crops during slow-growth conservation, which typically involves tissue culture. Molecular markers have confirmed that all genotypes preserved in a defined in vitro slow-growth conservation medium are genetically stable and pure. For instance, plantlets of *Dendrobium nobile* and *Saccharum officinarum*, regenerated from their calli, exhibited no genetic variation [31, 32]. Teeluck et al. [33] reported 1.9% genetic variation and 98.2% genetic fidelity among *Artocarpus altilis* callus-regenerated plantlets, demonstrating that ISSR markers are effective for detecting genetic variation. In this context; ILP markers provide a reliable, cost-effective, and user-friendly method for measuring genetic fidelity, with abundant polymorphism. Numerous studies on various crops, such as onion (*Allium cepa* L.) and carrot (*Daucus carota* L.) [34], have successfully used these markers to assess genetic fidelity within species [35].The current study further confirmed that slow-growing plantlets, conserved at ambient temperature (25 °C ± 2 °C), did not show any detrimental effects on the DNA or protein integrity of the surviving plants.

## Conclusions

While various studies have explored the conservation of *Allium* species, particularly garlic, with a focus on cryopreservation and long-term conservation [9–11], the present research provides a fresh and complementary perspective by focusing on conservation under ambient temperature conditions. The standardized protocol developed in this study is cost-effective and efficient, offering a viable alternative to cryopreservation and extensive field maintenance. This minimal-growth conservation regimen requires only two sub-culturing cycles per year, making it a practical and scalable solution for garlic conservation. Notably, this study is the first to examine the slow-growth conservation of diverse garlic genotypes at ambient room temperature. The method described here is simple, practical, and cost-effective, making it applicable to various cultivars and ecotypes. It contributes to advancing our understanding of slow-growth conservation strategies, particularly for bulbous crops like garlic.

The findings from this research have direct implications for large-scale conservation of garlic genotypes and can significantly enhance conservation efforts. This approach is both reliable and genotype-independent, offering a valuable strategy for long-term storage and in vitro observation. It effectively addresses challenges in genetic conservation and germplasm management, providing a robust framework for preserving garlic diversity.

## Supplementary Information

Supplementary Material 1.

Supplementary Material 2.

Supplementary Material 3.

Supplementary Material 4.

Supplementary Material 5.

Supplementary Material 6.

Supplementary Material 7.

Supplementary Material 8.

Supplementary Material 9.

Supplementary Material 10.

Supplementary Material 11.

## Data Availability

All the data and supplementary materials related to this article have been provided in the paper and used to accomplish the study goal. On request from the relevant author, raw data used in statistical analyses is accessible for future usage.
