# Supplementary material for: Optimizing in vitro slow-growth conservation media for garlic under ambient conditions: further implications for core set accessions
Source: BMC Plant Biol. 2025 Aug 4;25:1022. doi: 10.1186/s12870-025-06892-1 (PMC12320307; doi:10.1186/s12870-025-06892-1)
Supplement: Supplementary file 1 — Supplementary Material 1. [file 12870_2025_6892_MOESM1_ESM.docx]

Figure S1. Effect of osmoticums on garlic *in vitro* plantlets over conservation period for survival rate (%)
