# Supplementary figures and images for: Optimizing in vitro slow-growth conservation media for garlic under ambient conditions: further implications for core set accessions
Source: BMC Plant Biol. 2025 Aug 4;25:1022. doi: 10.1186/s12870-025-06892-1 (PMC12320307; doi:10.1186/s12870-025-06892-1)

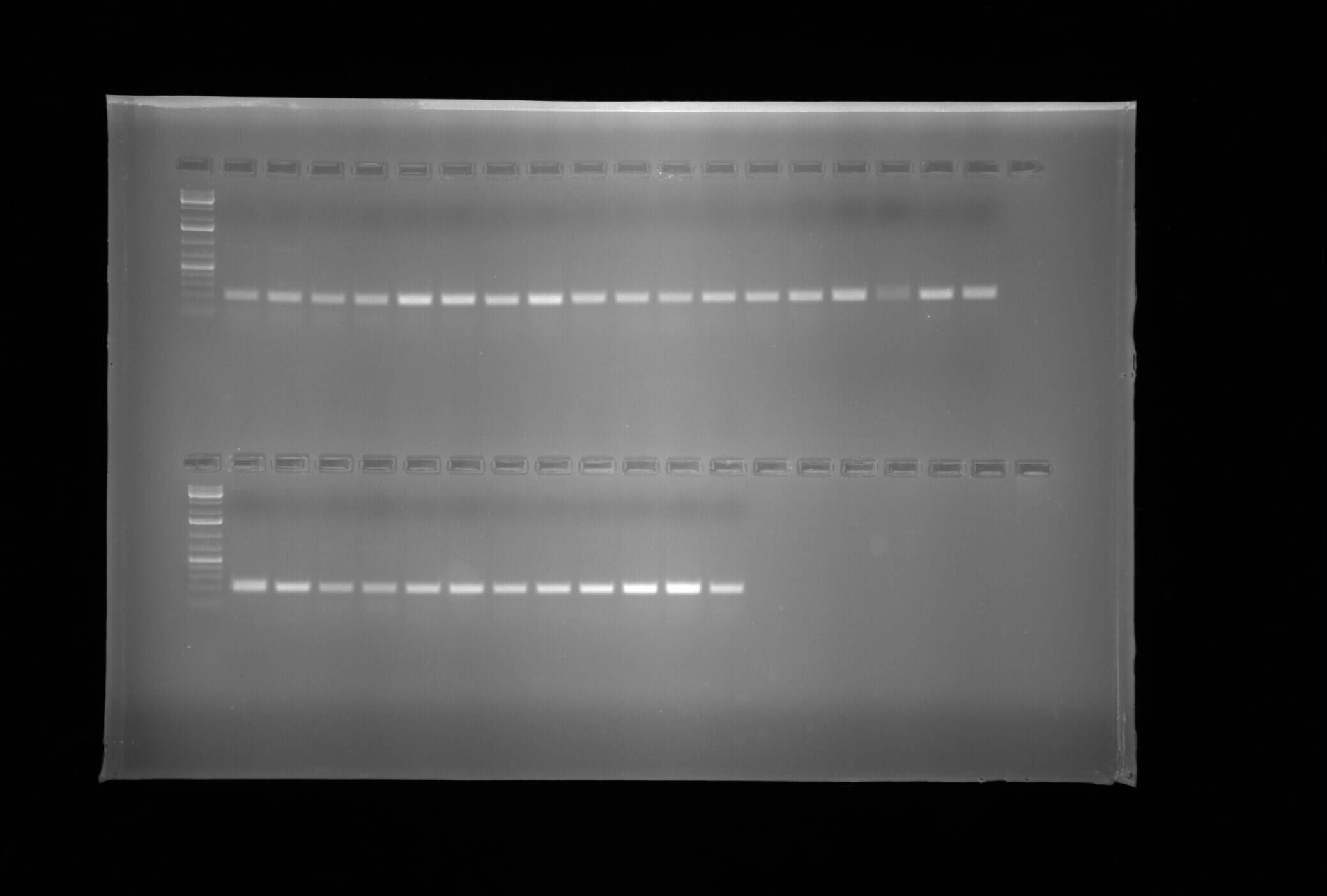

Supplement: Supplementary file 2 — Supplementary Material 2. [file 12870_2025_6892_MOESM2_ESM.jpg]

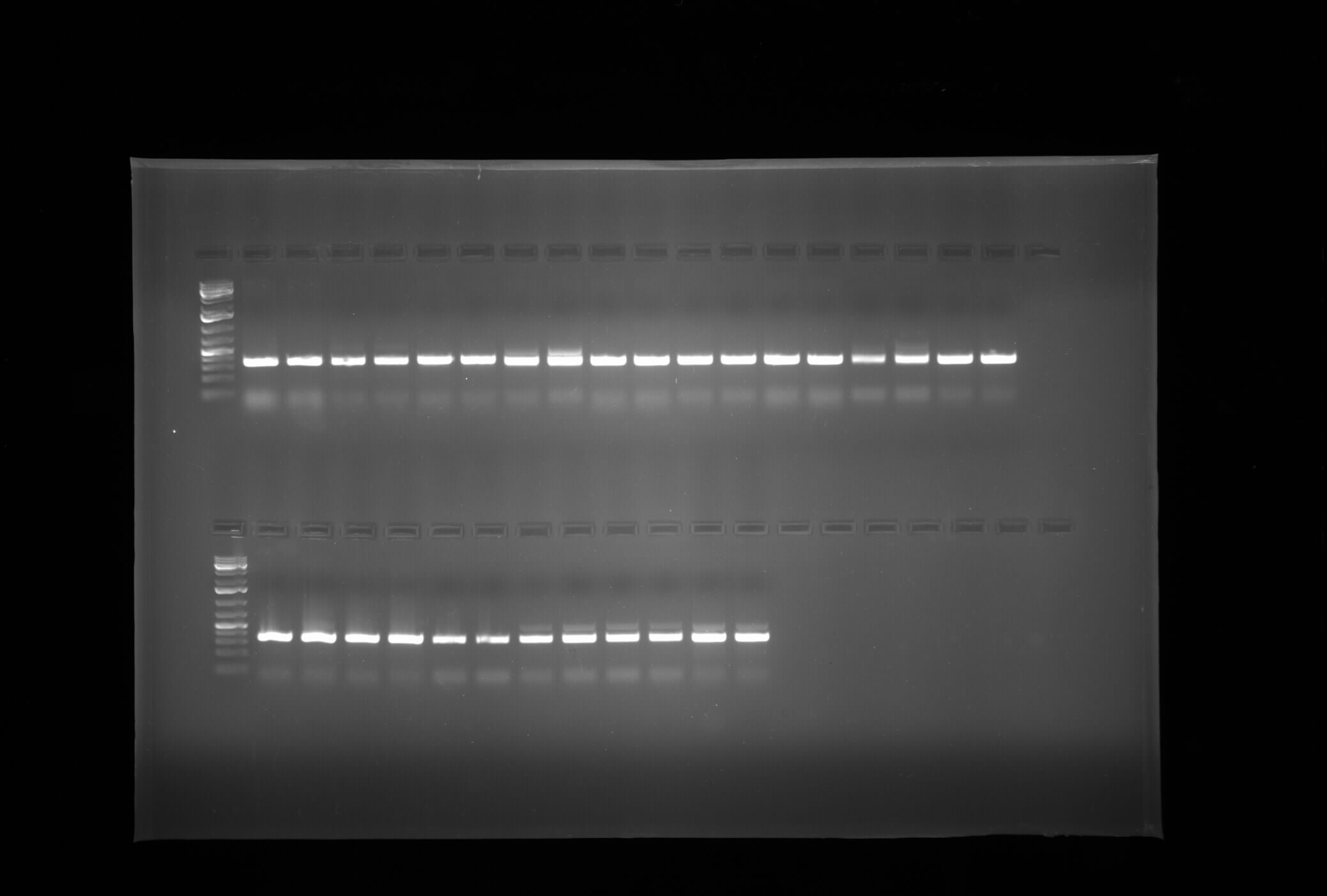

Supplement: Supplementary file 3 — Supplementary Material 3. [file 12870_2025_6892_MOESM3_ESM.jpg]

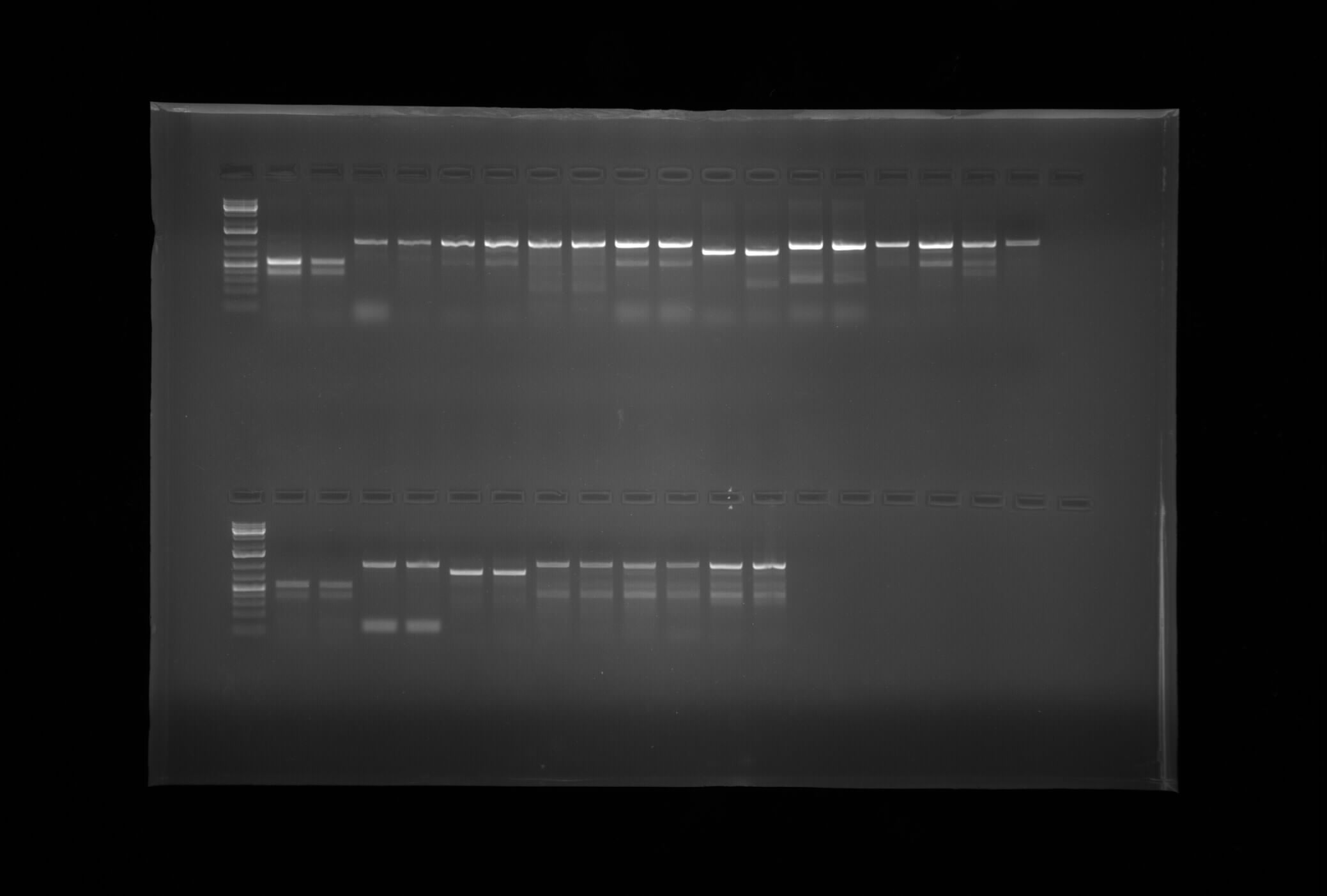

Supplement: Supplementary file 4 — Supplementary Material 4. [file 12870_2025_6892_MOESM4_ESM.jpg]
