# Supplementary material for: Optimizing in vitro slow-growth conservation media for garlic under ambient conditions: further implications for core set accessions
Source: BMC Plant Biol. 2025 Aug 4;25:1022. doi: 10.1186/s12870-025-06892-1 (PMC12320307; doi:10.1186/s12870-025-06892-1)
Supplement: Supplementary file 5 — Supplementary Material 5. [file 12870_2025_6892_MOESM5_ESM.docx]

Supplementary Table 1 : Treatment details of osmotic agents

| **Treatment** | **Details** | **Treatment** | **Details** |
| --- | --- | --- | --- |
| Treatment 1 | Control | Treatment 12 | 2 % Sucrose+ 2 % Sorbitol |
| Treatment 2 | Sucrose 1 % | Treatment 13 | 2 % Sucrose+ 4 %Sorbitol |
| Treatment 3 | Sucrose 2 % | Treatment 14 | 3 % Sucrose+ 2 % Sorbitol |
| Treatment 4 | Sucrose 3 % | Treatment 15 | 3 % Sucrose+4 % Sorbitol |
| Treatment 5 | Sucrose 4 % | Treatment 16 | 4 % Sucrose+ 2 % Sorbitol |
| Treatment 6 | Sorbitol 2 % | Treatment 17 | 4 % Sucrose+ 4 % Sorbitol |
| Treatment 7 | Sorbitol 4 % | Treatment 18 | 1 % Sucrose+2 % Mannitol |
| Treatment 8 | Mannitol 2 % | Treatment 19 | 1 % Sucrose+ 4 % Mannitol |
| Treatment 9 | Mannitol 4 % | Treatment 20 | 2 % Sucrose+ 2 % Mannitol |
| Treatment 10 | 1% Sucrose + 2 % Sorbitol | Treatment 21 | 2 % Sucrose+ 4 % Mannitol |
| Treatment 11 | 1% Sucrose+ 4 % Sorbitol |  |  |
