# Supplementary material for: Optimizing in vitro slow-growth conservation media for garlic under ambient conditions: further implications for core set accessions
Source: BMC Plant Biol. 2025 Aug 4;25:1022. doi: 10.1186/s12870-025-06892-1 (PMC12320307; doi:10.1186/s12870-025-06892-1)
Supplement: Supplementary file 6 — Supplementary Material 6. [file 12870_2025_6892_MOESM6_ESM.docx]

Table S3: List of 46 garlic core set accessions used for studying the effect of identified slow growth media combination.

| **Sr No.** | **DOGR Code** | **Accession No** | **Original site of Collection** |
| --- | --- | --- | --- |
| 1 | 18 | 650 | Odisha |
| 2 | 448 | 549 | Andhra Pradesh |
| 3 | 123 | IC-375119 | Haryana |
| 4 | 357 | IC-375056 | New Delhi |
| 5 | 294 | IC-372995 | Gujarat |
| 6 | 220 | EC-631741(594) | J&K |
| 7 | 367 | WG-432 | Andhra Pradesh |
| 8 | 20 | RG-95 | New Delhi |
| 9 | 374 | IC-14138 | New Delhi |
| 10 | 355 | IC-372921 | New Delhi |
| 11 | 543 | 543 Variety | Gujarat |
| 12 | 437 | IC-141310 | Uttaranchal |
| 13 | 540 | GG-4 | Gujarat |
| 14 | 291 | WG-418 | Andhra Pradesh |
| 15 | 561 | G-50 | Maharashtra |
| 16 | 486 | 520 | West Bengal |
| 17 | 571 | IC-372947 | Gujarat |
| 18 | 365 | WG-80 | Tamil Nadu |
| 19 | 436 | WG-23 | Maharashtra |
| 20 | 266 | NO-15 | Maharashtra |
| 21 | 319 | RG-343 | Maharashtra |
| 22 | 583 | G-282 | Maharashtra |
| 23 | 267 | 645 | Odisha |
| 24 | 542 | IC-141325 | New Delhi |
| 25 | 258 | IC-372924 | Gujarat |
| 26 | 595 | IC-32274 | New Delhi |
| 27 | 176 | 593 | J&K |
| 28 | 534 | WG-101 | Rajasthan |
| 29 | 110 | IC-322975 | New Delhi |
| 30 | 432 | 644 | Odisha |
| 31 | 318 | IC-338618 | Uttaranchal |
| 32 | 32 | WG-38 | Gujarat |
| 33 | 104 | RG-61 | Gujarat |
| 34 | 214 | M-199 | Haryana |
| 35 | 366 | IC-375087 | Gujarat |
| 36 | 456 | IC-48651 | New Delhi |
| 37 | 94 | IC-32881 | New Delhi |
| 38 | 28 | IC-34582 | Uttaranchal |
| 39 | 148 | IC-375028 | Gujarat |
| 40 | 339 | M-176 | Rajasthan |
| 41 | 538 | 644 | Odisha |
| 42 | 488 | 549 | Andhra Pradesh |
| 43 | 161 | M-90 | New Delhi |
| 44 | 401 | WG-14 | Gujarat |
| 45 | 50 | M-343 | Maharashtra |
| 46 | 570 | AC-200 | Bihar |
