# Supplementary material for: Optimizing in vitro slow-growth conservation media for garlic under ambient conditions: further implications for core set accessions
Source: BMC Plant Biol. 2025 Aug 4;25:1022. doi: 10.1186/s12870-025-06892-1 (PMC12320307; doi:10.1186/s12870-025-06892-1)
Supplement: Supplementary file 7 — Supplementary Material 7. [file 12870_2025_6892_MOESM7_ESM.docx]

Table S4: Mean performance of growth parameters of garlic plantlets under slow growth conservation (*in vitro*)

| **Sr No.** | **ACC. No.** | **No of Roots** | **Shoot length *** | **Plant Status** | **Mortality (%)** | **Group** |
| --- | --- | --- | --- | --- | --- | --- |
|  | 561 | 3 | 3 | 2.67 | 5.11 | First group |
|  | 339 | 2.67 | 2.00 | 2.33 | 6.58 |  |
|  | 291 | 3.00 | 2.33 | 3.00 | 5.99 |  |
|  | 318 | 3.00 | 2.33 | 2.33 | 5.88 |  |
|  | 488 | 3.00 | 3.00 | 2.67 | 6.15 |  |
|  | 266 | 3.00 | 3.00 | 3.00 | 9.65 |  |
|  | 367 | 3.00 | 3.00 | 3.00 | 6.58 |  |
|  | 543 | 2.00 | 2.67 | 2.33 | 7.21 |  |
|  | 357 | 2.67 | 2.33 | 2.67 | 7.22 |  |
|  | 176 | 3.00 | 2.67 | 3.00 | 9.12 |  |
|  | 161 | 2.67 | 3.00 | 2.67 | 8.54 |  |
|  | 267 | 2.33 | 2.67 | 2.33 | 8.14 |  |
|  | 220 | 2.33 | 2.67 | 1.67 | 6.88 |  |
|  | 374 | 2.33 | 2.33 | 2.67 | 7.56 |  |
|  | 365 | 2.67 | 3.00 | 2.67 | 9.51 |  |
|  | 583 | 2.67 | 2.67 | 2.33 | 8.69 |  |
|  | 570 | 2.67 | 2.00 | 2.67 | 9.54 |  |
|  | 595 | 2.67 | 3.00 | 2.00 | 6.58 |  |
|  | 104 | 2.33 | 2.67 | 1.67 | 8.25 |  |
|  | 94 | 0.33 | 0.33 | 2.00 | 15.21 | Second Group |
|  | 20 | 1.67 | 1.33 | 1.33 | 18.69 |  |
|  | 148 | 2.00 | 1.00 | 1.67 | 17.56 |  |
|  | 432 | 1.33 | 2.00 | 1.67 | 14.58 |  |
|  | 448 | 1.33 | 2.00 | 1.67 | 12.35 |  |
|  | 534 | 1.33 | 2.00 | 1.33 | 13.14 |  |
|  | 319 | 2.33 | 1.33 | 1.33 | 11.12 |  |
|  | 486 | 1.67 | 2.00 | 1.67 | 12.35 |  |
|  | 258 | 1.33 | 0.33 | 1.67 | 18.24 |  |
|  | 436 | 0.33 | 1.33 | 1.00 | 16.24 |  |
|  | 110 | 1.00 | 2.00 | 1.67 | 15.24 |  |
|  | 437 | 1.67 | 1.00 | 1.33 | 20.14 |  |
|  | 294 | 2.33 | 1.67 | 1.33 | 16.21 |  |
|  | 18 | 2.33 | 1.33 | 1.33 | 20.11 |  |
|  | 366 | 1.67 | 2.00 | 1.33 | 16.58 |  |
|  | 32 | 2.00 | 1.67 | 2.33 | 50.24 | Third Group |
|  | 28 | 1.00 | 1.33 | 2.33 | 40.21 |  |
|  | 538 | 2.00 | 1.67 | 2.00 | 34.26 |  |
|  | 542 | 1.33 | 2.00 | 2.67 | 32.34 |  |
|  | 456 | 2.00 | 1.67 | 2.33 | 22.25 |  |
|  | 200 | 1.33 | 2.33 | 3.00 | 21.23 |  |
|  | 571 | 2.00 | 1.67 | 2.33 | 26.24 |  |
|  | 214 | 1.67 | 2.00 | 2.33 | 24.28 |  |
|  | 540 | 2.00 | 1.33 | 2.67 | 32.46 |  |
|  | 123 | 1.33 | 2.00 | 2.33 | 36.25 |  |
|  | 502 | 1.34 | 2.13 | 2.33 | 22.58 |  |
|  | 355 | 1.36 | 2.15 | 2.56 | 26.24 |  |

***(transformed values)**
