# Supplementary material for: Optimizing in vitro slow-growth conservation media for garlic under ambient conditions: further implications for core set accessions
Source: BMC Plant Biol. 2025 Aug 4;25:1022. doi: 10.1186/s12870-025-06892-1 (PMC12320307; doi:10.1186/s12870-025-06892-1)
Supplement: Supplementary file 8 — Supplementary Material 8. [file 12870_2025_6892_MOESM8_ESM.docx]

**Table S2b.** Effect of different osmotic concentrations on growth parameters of garlic plantlets at third month (90 days to culture) of *in-vitro* slow growth conservation.

| **Sr no** | **Treatment combinations** | **Parameters recorded at 3^rd^ month of slow growth conservation** | | | | | |
| --- | --- | --- | --- | --- | --- | --- | --- |
|  |  | Shoot length (cm) | Root length (cm) | Number of leaves | Number of roots | Plant status | Survival (%) |
|  | Control | 7.43 ef | 0.31 j | 2.149bdec | 2.86 edf | 1.22e | 0 f |
|  | 1% Sucrose | 15.57 a | 7.71 ebdac | 3.43 ba | 3.00 ebdac | 1.3129ed | 28 de |
|  | 2 % Sucrose | 13.79 a | 3.79 edfc | 3.00 bac | 3.00 bac | 1.3129ed | 28 de |
|  | 3% Sucrose | 13.29 abcd | 5.04 ebdac | 3.43 a | 3.00 bdac | 1.5757dc | 34 d |
|  | 4% Sucrose | 13.86 f | 6.71 gfh | 2.43 jih | 3.57 ebdac | 1.565dc | 28 de |
|  | 2% Sorbitol | 9.43 bdc | 3.50 gfh | 3.43 bdac | 2.00 edfc | 1.7664bc | 50 bac |
|  | 4%Sorbitol | 7.14 e | 2.43 gfh | 1.14 gjih | 1.60 edf | 1.9921ba | 80 a |
|  | 2% Mannitol | 2.29 f | 0.45 ji | 1.14 jk | 1.50 ef | 1.9507ba | 85 ba |
|  | 4% Mannitol | 2.86 g | 2.21 ih | 1.00 k | 0.61 f | 1.9743ba | 65 ba |
|  | 1% Sucrose + 2% Sorbitol | 12.79 ab | 2.47 gfh | 2.00 fgdeih | 2.43 bac | 1.9143ba | 65 ba |
|  | 1% Sucrose+ 4% Sorbitol | 9.71 cbd | 1.23 gh | 2.00 fgdch | 1.14 edfc | 1.8279bac | 100 a |
|  | 2% Sucrose+ 2% Sorbitol | 11.71 abc | 3.74 egdf | 2.14 fbdec | 1.43 ebdc | 1.9764ba | 85 ba |
|  | 2% Sucrose+ 4%Sorbitol | 10.43 dc | 2.05 egf | 1.86 fdec | 2.43 ba | 2.0607ba | 100 a |
|  | 3% Sucrose+ 2% Sorbitol | 11.71 abcd | 5.86 ba | 2.00 fbdec | 2.00 ba | 2.0693ba | 100 a |
|  | 3% Sucrose+4% Sorbitol | 10.86 abcd | 5.30 bdac | 2.29 fgjeih | 2.67 ba | 1.9371ba | 85 ba |
|  | 4% Sucrose+ 2% Sorbitol | 10.71 abcd | 3.43 edfc | 2.14 bdec | 1.29 ebdac | 1.9521ba | 100 a |
|  | 4% Sucrose+ 4% Sorbitol | 10.14 d | 3.93 ebdfc | 1.86 fgdec | 1.71 bac | 2.0879a | 85 ba |
|  | 1% Sucrose+2% Mannitol | 13.00 a | 5.14 ebdac | 1.43 gjih | 2.71 bac | 1.8479bac | 71 bac |
|  | 1% Sucrose+ 4% Mannitol | 11.86 bcd | 7.43 a | 1.71 ji | 2.43 bac | 2.0164ba | 85 ba |
|  | 2% Sucrose+ 2% Mannitol | 12.14 abcd | 5.71 bdac | 1.57 ji | 2.57 bac | 1.9107ba | 71 bac |
|  | 2% Sucrose+4% Mannitol | 12.86 abc | 5.71 bac | 1.86 fgjih | 2.57 a | 1.8286bac | 71 bac |

(Means with same letter (s) are not significantly different)
