# Supplementary material for: Optimizing in vitro slow-growth conservation media for garlic under ambient conditions: further implications for core set accessions
Source: BMC Plant Biol. 2025 Aug 4;25:1022. doi: 10.1186/s12870-025-06892-1 (PMC12320307; doi:10.1186/s12870-025-06892-1)
Supplement: Supplementary file 9 — Supplementary Material 9. [file 12870_2025_6892_MOESM9_ESM.docx]

**Table S 1a:** Analysis of variance and means of different growth parameters of *in vitro* slow growth conserved garlic plantlets at 6^th^ month of conservation

| The SAS System | | |
| --- | --- | --- |
| The GLM Procedure | | |
| **Class Level Information** | | |
| **Class** | **Levels** | **Values** |
| **Replication** | 7 | 1 2 3 4 5 6 7 |
| **Varieties** | 2 | 1 2 (Bhima Purple, Bhima Omkar) |
| **Treatments** | 21 | 1 2 3 4 5 6 7 8 9 10 11 12 13 14 15 16 17 18 19 20 21 |
| **Shoot Length (cm)** | 16 | 0.71 2.55 2.74 2.92 3.08 3.24 3.39 3.54 3.58 3.67 3.81 3.94 4.06 4.18 4.3 4.42 |
| **Root Length (cm)** | 14 | 0.71 1.22 1.58 1.87 2.12 2.35 2.55 2.74 2.92 3.08 3.24 3.39 3.54 3.67 |
| **Number of Shoots** | 5 | 0.71 1.22 1.58 1.87 2.12 |
| **Number of Roots** | 7 | 0.71 1.22 1.58 1.87 2.12 2.35 2.55 |
| **Survival Percentage (%)** | 5 | 1.22 1.58 1.87 2.12 2.35 |
| **Number of Observations Read** | 294 |  |
| **Number of Observations Used** | 294 |  |

| Dependent Variable: shoot length (sl) (cm) | | | | | |
| --- | --- | --- | --- | --- | --- |
| **Source** | **DF** | **Sum of Squares** | **Mean Square** | **F Value** | **Pr > F** |
| **Model** | 41 | 410.787 | 10.0192 | 53.87 | <.0001 |
| **Error** | 252 | 46.869 | 0.18599 |  |  |
| **Corrected Total** | 293 | 457.656 |  |  |  |
|  | | | | | |
| **R-Square** | **Coeff Var** | **Root MSE** | **sl Mean** |  |  |
| 0.89759 | 14.3867 | 0.43126 | 2.99765 |  |  |
|  | | | | | |
| **Source** | **DF** | **Type I SS** | **Mean Square** | **F Value** | **Pr > F** |
| **Variety** | 1 | 2.34732 | 2.34732 | 12.62 | 0.0005 |
| **Treatment** | 20 | 339.431 | 16.9715 | 91.25 | <.0001 |
| **var*trt** | 20 | 69.0085 | 3.45042 | 18.55 | <.0001 |
|  | | | | | |
| **Source** | **DF** | **Type III SS** | **Mean Square** | **F Value** | **Pr > F** |
| **Variety** | 1 | 2.34732 | 2.34732 | 12.62 | 0.0005 |
| **Treatment** | 20 | 339.431 | 16.9715 | 91.25 | <.0001 |
| **var*trt** | 20 | 69.0085 | 3.45042 | 18.55 | <.0001 |

| Dependent Variable: Root length (cm) | | | | | |
| --- | --- | --- | --- | --- | --- |
| **Source** | **DF** | **Sum of Squares** | **Mean Square** | **F Value** | **Pr > F** |
| **Model** | 41 | 186.291 | 4.54367 | 33.86 | <.0001 |
| **Error** | 252 | 33.8162 | 0.13419 |  |  |
| **Corrected Total** | 293 | 220.107 |  |  |  |
|  | | | | | |
| **R-Square** | **Coeff Var** | **Root MSE** | **Root length Mean** |  |  |
| 0.84637 | 16.8008 | 0.36632 | 2.18037 |  |  |
|  | | | | | |
| **Source** | **DF** | **Type I SS** | **Mean Square** | **F Value** | **Pr > F** |
| **Variety** | 1 | 4.36176 | 4.36176 | 32.5 | <.0001 |
| **Treatment** | 20 | 135.617 | 6.78086 | 50.53 | <.0001 |
| **var*trt** | 20 | 46.3116 | 2.31558 | 17.26 | <.0001 |
|  | | | | | |
| **Source** | **DF** | **Type III SS** | **Mean Square** | **F Value** | **Pr > F** |
| **Variety** | 1 | 4.36176 | 4.36176 | 32.5 | <.0001 |
| **Treatment** | 20 | 135.617 | 6.78086 | 50.53 | <.0001 |
| **var*trt** | 20 | 46.3116 | 2.31558 | 17.26 | <.0001 |

| Dependent Variable: Number of shoots (ns) | | | | | |
| --- | --- | --- | --- | --- | --- |
|  | | | | | |
| **Source** | **DF** | **Sum of Squares** | **Mean Square** | **F Value** | **Pr > F** |
| **Model** | 41 | 28.5861 | 0.69722 | 16.79 | <.0001 |
| **Error** | 252 | 10.4638 | 0.04152 |  |  |
| **Corrected Total** | 293 | 39.0499 |  |  |  |
|  | | | | | |
| **R-Square** | **Coeff Var** | **Root MSE** | **ns Mean** |  |  |
| 0.73204 | 16.3173 | 0.20377 | 1.24881 |  |  |
|  | | | | | |
| **Source** | **DF** | **Type I SS** | **Mean Square** | **F Value** | **Pr > F** |
| **Variety** | 1 | 0.72603 | 0.72603 | 17.48 | <.0001 |
| **Treatment** | 20 | 20.8235 | 1.04117 | 25.07 | <.0001 |
| **var*trt** | 20 | 7.03654 | 0.35183 | 8.47 | <.0001 |
|  | | | | | |
| **Source** | **DF** | **Type III SS** | **Mean Square** | **F Value** | **Pr > F** |
| **Variety** | 1 | 0.72603 | 0.72603 | 17.48 | <.0001 |
| **Treatment** | 20 | 20.8235 | 1.04117 | 25.07 | <.0001 |
| **var*trt** | 20 | 7.03654 | 0.35183 | 8.47 | <.0001 |

| Dependent Variable: Number of roots (nr) | | | | | | | |
| --- | --- | --- | --- | --- | --- | --- | --- |
|  | | | | | | | |
| **Source** | **DF** | **Sum of Squares** | | **Mean Square** | | **F Value** | **Pr > F** |
| **Model** | 41 | 75.4875 | | 1.84116 | | 31.04 | <.0001 |
| **Error** | 252 | 14.9467 | | 0.05931 | |  |  |
| **Corrected Total** | 293 | 90.4342 | |  | |  |  |
|  | | | | | | | |
| **R-Square** | **Coeff Var** | | **Root MSE** | **nr Mean** |  | |  |
| 0.83472 | 15.0949 | | 0.24354 | 1.6134 |  | |  |
|  | | | | | | | |
| **Source** | **DF** | **Type I SS** | | **Mean Square** | **F Value** | | **Pr > F** |
| **Variety** | 1 | 1.66427 | | 1.66427 | 28.06 | | <.0001 |
| **Treatment** | 20 | 65.9231 | | 3.29615 | 55.57 | | <.0001 |
| **var*trt** | 20 | 7.90022 | | 0.39501 | 6.66 | | <.0001 |
|  | | | | | | | |
| **Source** | **DF** | **Type III SS** | | **Mean Square** | **F Value** | | **Pr > F** |
| **Variety** | 1 | 1.66427 | | 1.66427 | 28.06 | | <.0001 |
| **Treatment** | 20 | 65.9231 | | 3.29615 | 55.57 | | <.0001 |
| **var*trt** | 20 | 7.90022 | | 0.39501 | 6.66 | | <.0001 |

| Dependent Variable: Survival Rate (%) (s%) | | | | | |
| --- | --- | --- | --- | --- | --- |
|  |  |  |  |  |  |
| **Source** | **DF** | **Sum of Squares** | **Mean Square** | **F Value** | **Pr > F** |
| **Model** | 41 | 28.0495 | 0.68413 | 10.76 | <.0001 |
| **Error** | 252 | 16.0231 | 0.06358 |  |  |
| **Corrected Total** | 293 | 44.0726 |  |  |  |
|  | | | | | |
| **R-Square** | **Coeff Var** | **Root MSE** | **s % Mean** |  |  |
| 0.63644 | 15.775 | 0.25216 | 1.59847 |  |  |
|  | | | | | |
| **Source** | **DF** | **Type I SS** | **Mean Square** | **F Value** | **Pr > F** |
| **Variety** | 1 | 0.10477 | 0.10477 | 1.65 | 0.2004 |
| **Treatment** | 20 | 22.0242 | 1.10121 | 17.32 | <.0001 |
| **var*trt** | 20 | 5.92057 | 0.29603 | 4.66 | <.0001 |
|  | | | | | |
| **Source** | **DF** | **Type III SS** | **Mean Square** | **F Value** | **Pr > F** |
| **Variety** | 1 | 0.10477 | 0.10477 | 1.65 | 0.2004 |
| **Treatment** | 20 | 22.0242 | 1.10121 | 17.32 | <.0001 |
| **var*trt** | 20 | 5.92057 | 0.29603 | 4.66 | <.0001 |

*DF- Degree of freedom
