# Supplementary material for: Optimizing in vitro slow-growth conservation media for garlic under ambient conditions: further implications for core set accessions
Source: BMC Plant Biol. 2025 Aug 4;25:1022. doi: 10.1186/s12870-025-06892-1 (PMC12320307; doi:10.1186/s12870-025-06892-1)
Supplement: Supplementary file 10 — Supplementary Material 10. [file 12870_2025_6892_MOESM10_ESM.docx]

**Table S2a.** Effect of different osmotic concentrations on morphological traits of garlic at first month (30 Days to Culture) of *in-vitro* conservation.

| **Sr no** | **Treatment combinations** | **Parameters recorded at 1^st^ month of slow growth conservation** | | | | | |
| --- | --- | --- | --- | --- | --- | --- | --- |
|  |  | Shoot length (cm) | Root length (cm) | Number of leaves | Plant status | Number of roots | Survival (%) |
| 1 | Control | 5.93 ef | 0.23 j | 2.14 bdec | 1.22e | 2.33 edf | 0 f |
| 2 | 1% Sucrose | 13.71 a | 7.71 ebdac | 3.14 ba | 1.3129ed | 3.00 ebdac | 28 de |
| 3 | 2 % Sucrose | 13.64 a | 3.63 edfc | 2.86 bac | 1.3129ed | 2.29 bac | 28 de |
| 4 | 3% Sucrose | 11.93 abcd | 5.66 ebdac | 2.43 a | 1.5757dc | 2.40 bdac | 34 d |
| 5 | 4% Sucrose | 12.71 f | 6.83 gfh | 2.14 jih | 1.565dc | 2.20 ebdac | 28 de |
| 6 | 2% Sorbitol | 8.71 bdc | 3.50 gfh | 2.14 bdac | 1.7664bc | 1.00 edfc | 64 bac |
| 7 | 4%Sorbitol | 5.71 e | 5.00 gfh | 1.14 gjih | 1.9921ba | 1.00 edf | 80 a |
| 8 | 2% Mannitol | 1.46 f | 0.30 ji | 1.14 jk | 1.9507ba | 1.00 ef | 60 ba |
| 9 | 4% Mannitol | 0.67 g | 0.00 ih | 1.00 k | 1.9743ba | 0.00 f | 71 ba |
| 10 | 1% Sucrose + 2% Sorbitol | 11.64 ab | 2.62 gfh | 2.00 fgdeih | 1.9143ba | 1.67 bac | 85 ba |
| 11 | 1% Sucrose+ 4% Sorbitol | 8.43 cbd | 2.00 gh | 2.00fgd | 1.8279bac | 1.00 edfc | 100 a |
| 12 | 2% Sucrose+ 2% Sorbitol | 11.29 abc | 5.33 egdf | 2.14 fbdec | 1.9764ba | 1.33 ebdc | 85 ba |
| 13 | 2% Sucrose+ 4%Sorbitol | 9.86 dc | 3.33 egf | 2.14 fdec | 2.0607ba | 1.67 ba | 100 a |
| 14 | 3% Sucrose+ 2% Sorbitol | 11.14 abcd | 6.40 ba | 2.00 fbdec | 2.0693ba | 1.40 ba | 100 a |
| 15 | 3% Sucrose+4% Sorbitol | 10.29 abcd | 0.20 bdac | 2.29 fgjeih | 1.9371ba | 1.00 ba | 100 ba |
| 16 | 4% Sucrose+ 2% Sorbitol | 10.43 abcd | 5.001edfc | 2.14 bdec | 1.9521ba | 1.33 ebdac | 100 a |
| 17 | 4% Sucrose+ 4% Sorbitol | 9.29 d | 5.50 ebdfc | 1.86 fgdec | 2.0879a | 2.50 bac | 85 ba |
| 18 | 1% Sucrose+2% Mannitol | 7.57 a | 1.25 ebdac | 1.14 gjih | 1.8479bac | 1.50 bac | 85 bac |
| 19 | 1% Sucrose+ 4% Mannitol | 7.00 bcd | 2.00 a | 1.00 ji | 2.0164ba | 1.50 bac | 85 ba |
| 20 | 2% Sucrose+ 2% Mannitol | 4.43 abcd | 1.25 bdac | 1.29 ji | 1.9107ba | 2.00 bac | 71 bac |
| 21 | 2% Sucrose+4% Mannitol | 5.14 abc | 2.00 bac | 1.00 fgjih | 1.8286bac | 1.50 a | 71 bac |

(Means with same letter (s) are not significantly different)
